# Class imbalance aware deep semantic segmentation framework for weed and tobacco crops in UAV imagery

**DOI:** 10.3389/frai.2026.1792086

**Published:** 2026-06-04

**Authors:** Ahmad Jaffar Khan, Muhammad Abu Bakr, Sultan Daud Khan, Habib Ullah, Mohib Ullah

**Affiliations:** 1 Department of Electrical Engineering, National University of Technology, Islamabad, Pakistan; 2 Faculty of Computing and Information Technology, Sohar University, Sohar, Oman; 3 Faculty of Science and Technology, Norwegian University of Life Sciences, Ås, Norway; 4 Department of Computer Science, Norwegian University of Science and Technology (NTNU), Gjøvik, Norway

**Keywords:** class imbalance, deep learning, smart agriculture, UAV imagery, weed segmentation

## Abstract

For accurate pesticide application in precision farming, weeds and tobacco plants must be detected to efficiently apply pesticides to weedy areas. There is potential for automated, precise weed and tobacco detection using unmanned aerial vehicle (UAV)-based imaging. Semantic segmentation is a challenge that can be applied to accurately detect weeds in crop field images. Deep learning-based semantic segmentation techniques promise higher accuracy than prior approaches for pixel-level categorization in classical machine learning. In this study, we introduce a novel approach that enhances the accuracy of pixel-level crop–weed interclass classification. We suggested a DeepLabV3Plus ResNeSt model that was trained using the Lovász cross-entropy combined loss and inverse square root frequency weighted class using the tobacco weed UAV-based dataset, achieving a mean average accuracy (aAcc) of 95.93%, a mean intersection over union (mIoU) of 84.99%, and a mean accuracy (mAcc) score of 90.20%. Due to the limited number of pixels identified as weeds, the aerial images used constitute a limited dataset. Therefore, we adjusted class weights using the inverse square root frequency model, which simplified the segmentation process. We observed that the proposed model achieved the highest mean mIoU, indicating that it can accurately detect weeds and tobacco plants.

## 1 Introduction

Precision agriculture aims to maximize crop input according to needs, minimize environmental risk, and increase profitability (Karunathilake et al., 2023; Rebouh et al., 2023). Vegetation detection and segmentation are essential components of precision agriculture because they allow evaluation of crop health, the creation of variable prescription maps, early identification of crop issues, and the prompt preparation of treatments to reduce crop losses (Gupta et al., 2023). Unwanted plants, weeds in agricultural soil, constantly compete with crops for resources such as sunlight, nutrients, space, and water (Kumar et al., 2024). In addition to causing damage, weeds can make harvesting even more difficult. Indirectly, undesirable weeds will raise production costs, lower the quality of the product, increase the risk of pests and diseases, and lower the farmed area's economic value (Rao et al., 2020). It has been claimed that ineffective weed

control results in a yield reduction of 40% (Zhang et al., 2023). Therefore, one of the most important aspects of a cultivated field is weed detection, especially targeted herbicide spraying, which lowers the cost of chemical usage and management (Ehrampoosh et al., 2025).

Weed detection is now done through smart agriculture, a cutting-edge method that uses big data analysis, machine learning, and remote sensing (Jonak et al., 2024). The four basic steps of a common machine learning-based weed identification method are feature extraction, classification, image pre-processing, and image acquisition (Hasan et al., 2021). Red, green, and blue (RGB) or multispectral images are the most widely used image acquisition formats. These formats are obtained from images captured by cameras or sensors installed on various platforms, including field robots (Rani et al., 2022), all-terrain vehicles (Partel et al., 2019), unmanned aerial vehicles (UAVs) (Reedha et al., 2022), and even satellites (Martin et al., 2018).

For understanding visual content, segmentation is a crucial activity with multiple applications (Ronneberger et al., 2015; Cordts et al., 2016). A basic assignment in computer vision is to assign a class label to each pixel in an image. The segmentation methods that are used most frequently are learning-based segmentation with machine learning models (Zou et al., 2021), unsupervised clustering algorithms (Zhang et al., 2021), and threshold-based segmentation, such as Otsu's method (Wang et al., 2019). From the segmented images, several features are extracted, including shape, color, texture, and spectral properties. To separate weeds from crops and soil, the final classification phase often employs additional machine learning models, such as decision trees, random forests (RFs), support vector machines (SVMs), logistic regression, and k-nearest neighbors (KNN) (Lauwers et al., 2022; Islam et al., 2021; Yuba et al., 2021; Bakhshipour and Zareiforoush, 2020). These UAVs also generate a substantial volume of very high-resolution images for the previously stated uses. Therefore, deep learning techniques, such as convolutional neural networks (CNNs), have become crucial for remote sensing image/video processing, particularly for tackling tasks such as vegetation mapping and weed detection.

However, the extremely high-resolution images captured by UAVs may not be suitable for deep models developed for satellite imagery (Zhou et al., 2016). A comparison of UAVs and satellite imagery shows that UAVs provide greater semantic and geographical detail than satellite imagery (Shahi et al., 2023). It is possible for different objects to have a comparable color intensity. However, with UAV-based images, the job becomes more difficult because the identical items' width and length could vary across intra- and inter-images, among other spatial properties (Shahi et al., 2023; Sandoval-Pillajo et al., 2025). Due to this, building a strong system is essential for managing these tasks (Bhandari and Etienne, 2025).

The present study addresses this challenge by implementing a weed-detection framework that identifies weed pixels in UAV-captured images. It is achieved using the proposed improved DeepLabV3Plus ResNeSt deep learning architecture. The proposed model achieves significantly better performance with a Lovász cross-entropy combined loss and an inverse square root frequency weighted class. The experiments were conducted on a publicly available tobacco weed UAV-based image dataset (Moazzam et al., 2023). The main contributions of this study include the following:

1. To effectively separate weeds from tobacco plants in UAV images, an improved DeepLabV3Plus ResNeSt model is proposed, combining the Lovász cross-entropy loss with inverse square root frequency class weighting. This model improves intersection over union (IoU) and overall accuracy through tailored loss and weighting strategies.
2. Using the DeepLabV3Plus ResNeSt encoder and UAV imagery, an in-depth study was conducted on the effects of various loss and class-weighting functions. This analysis used a UAV tobacco-weed image dataset that covers pixel-level interclass classification. Weighted class functions were used to compensate for the small number of weed pixels in the UAV weed dataset.
3. Using a tobacco-weed UAV dataset, we thoroughly compared the performance of the proposed model with various pretrained semantic segmentation methods, demonstrating the significance of the proposed framework relative to benchmark approaches.

The remainder of this article is structured as follows. The literature review is covered in Section 2. A detailed description of the segmentation method, the dataset used, and the experimental configuration is given in Section 3. The experimental results and observations are covered in Section 4. The current study is summarized and concluded in Section 6.

## 2 Literature review

By systematically altering weed management methods on a farm to account for changes in weed population size, distribution, and diversity, Site-Specific Weed Management (SSWM) emerges as an innovative strategy (Somerville et al., 2020). The development of a weed geographic information map is the foundation of SSWM, acknowledging the irregular distribution of weed populations across farmlands. This map is an essential tool for accurately applying agrochemicals within a regulated system. Direct chemical application to targeted weeds is the goal, but alternative approaches, including plant-derived treatments that leverage allelopathic effects as natural weed killers, are also being integrated. By reducing soil, water, and air pollution, this lessens the ecological footprint, tackles weed issues, and reduces chemical contamination (Al-Samarai et al., 2018).

Traditional machine learning techniques have been employed by numerous researchers to detect weeds. Conventional machine learning-based methods employ machine learning-based classifiers for classification, detection, or segmentation, and feature descriptors to extract object attributes from sensory data. Using drone images taken from chili fields in Australia, machine learning algorithms such as random forest (RF), support vector machines (SVMs), and k-nearest neighbors (KNN) can detect weeds with high accuracy rates. The corresponding weed-detection accuracies for RF, SVM, and KNN were 96, 94, and 63%, respectively (Islam et al., 2021). An SVM classifier based on texture, shape, and color achieved up to 96% classification accuracy and 6 frames per second (FPS) detection speed (Khan et al., 2021). However, the popularity and application of conventional machine learning in weed detection are limited because these methods cannot extract features on their own; instead, they require manually designed features (such as color, location, and texture) (Espejo-Garcia et al., 2020).

Deep learning-based detection techniques have emerged as the most popular strategy in the field of weed identification in recent years (Espejo-Garcia et al., 2020; Liu and Bruch, 2020; Quan et al., 2021). Deep learning-based algorithms eliminate the challenging manual feature learning process, which is a crucial component of machine learning algorithms, especially when tackling rapidly expanding data. Methods influenced by deep learning, such as CNNs, are frequently employed for satellite and UAV-based remote sensing applications (Zhu et al., 2017). By applying the boundary-oriented binary building segmentation model proposed by Lee et al. (2021), it is possible to determine the boundaries of the segmented structures from aerial imagery.

Challenges with weed recognition include overlapping weeds and crops, too-dense recognition targets, and varying weed sizes and forms. Many studies have employed deep learning-based semantic segmentation algorithms for weed recognition to better address these issues. The ability of a deep learning-based semantic segmentation system to classify items pixel by pixel to identify weeds allows it to separate objects with irregular outlines and those that are densely distributed (Quan et al., 2021). The SegNet (Badrinarayanan et al., 2017), U-Net (Ronneberger et al., 2015), and DeepLab series models (Chen, 2014; Chen et al., 2017; Sandler et al., 2018) are the most widely utilized semantic segmentation models currently used for weed recognition tasks.

DeepLabV3Plus uses an encoder-decoder architecture with atrous separable convolutions, which helps extract multi-scale contextual features while preserving spatial details (Hashemi-Beni et al., 2022; Wang et al., 2024). This makes it especially useful for agricultural images, where crops and weeds can vary in size and how densely they are packed. Many recent studies have shown that DeepLabV3Plus works well for weed segmentation. For example, a version based on the Swin Transformer, called Swin-DeepLab, achieved an mIoU of 91.53% for weed detection in soybean fields (Yu et al., 2022). In images from maize fields, DeepLabV3Plus performed better than other architectures like U-Net, LinkNet, and FPN, achieving a mIoU of 76% for crop segmentation and allowing accurate estimation of weed density (Bak et al., 2023). A recent study has also explored lighter versions of DeepLabV3Plus to enable effective operation on drones and edge devices, using backbones such as MobileNetV2 and MobileNetV3 to improve computational efficiency without sacrificing segmentation performance (Islam et al., 2025). However, there are still challenges in using drones for weed segmentation, including insufficient annotated data, environmental changes, and the trade-off between accuracy and real-time processing.

Furthermore, the study shows that deep learning algorithms outperform basic machine learning methods in terms of extraction accuracy, such as SVM and RF. Many tasks related to aerial imagery segmentation are based on the fully convolutional structure (Ronneberger et al., 2015; Behera et al., 2023). In this framework, context information is extracted in one phase, and the resulting feature matrix is expanded to the original resolution of the input image in the next phase. Various architectures have also been developed by researchers to address the segmentation problems currently encountered in image segmentation tasks (Gibril et al., 2021; Pandey and Jain, 2022).

With UAV-based weed imaging, weed semantic segmentation is more challenging because the images are complex due to the large number of ground objects (Sandoval-Pillajo et al., 2025). However, UAV-based datasets are becoming more accessible and developed, thus more research is being done on weed semantic segmentation using these datasets (Zhao and Wang, 2025). Images from UAVs and satellites differ in terms of spatial and semantic detail. However, with UAV-based images, the task becomes more challenging, as spatial parameters such as the width and length of similar items can differ between intra- and inter-image pairs (Shahi et al., 2023). Therefore, a capable framework must be developed to handle this crucial task.

## 3 Materials and methods

**Figure 1:**
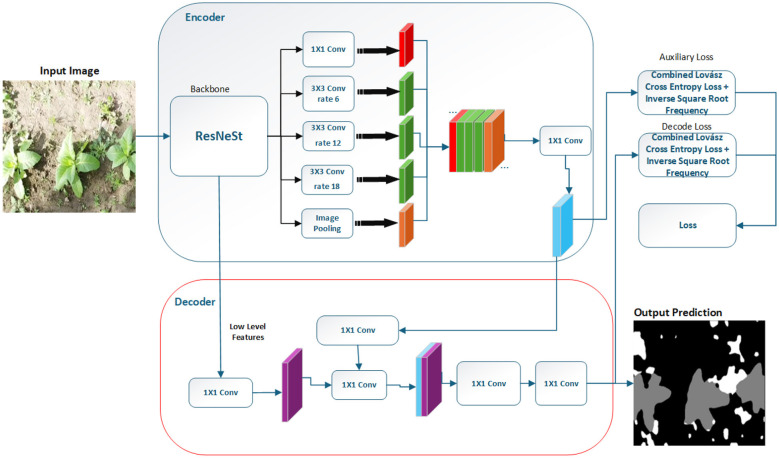
Encoder-decoder architecture of DeepLabV3Plus segmentation model with ResNeSt as backbone.

In this part, the segmentation process utilizing the improved DeepLabV3Plus with ResNeSt as the backbone network is briefly described. In addition to the utilization of various loss functions, evaluation metrics, class imbalance techniques, and a detailed description of the dataset used for this study, these are explained in the experimental setup. The improved DeepLabV3Plus with ResNeSt as the backbone proposed in this study for weed-detection segmentation is shown in Figure 1.

### 3.1 Improved DeepLabV3Plus with ResNeSt as backbone

Using an encoder-decoder architecture, the DeepLabV3Plus model improves segmentation while preserving the target's edge details (Wang et al., 2024). DeepLabV3 serves as the network's encoder, optimizing the extraction of target edge information. The decoder then recovers the feature information and outputs the predicted results. Using the Xception model as the backbone, DeepLabV3Plus applies deep separable convolutions to the decoder and Atrous Spatial Pyramid Pooling (ASPP) modules to produce an encoder-decoder network with improved segmentation performance. Xception uses inception-style designs and is largely based on effective modular components. However, it does not explicitly capture long-range dependencies between various feature map components.

To mitigate these drawbacks, our proposed improved DeepLabV3Plus model uses ResNeSt instead of Xception. ResNeSt uses Split Attention Networks, a ResNet variant, as its backbone and is created by stacking network blocks in the ResNet style (Zhang et al., 2022). The ResNeSt Split-Attention module helps the model learn more complex connections and patterns by improving its ability to capture multi-scale features and local context through cross-feature interactions. As it allows the network to better handle long-range relationships and gather more contextual information, this is especially helpful for tasks like segmentation. The ResNeSt findings are sent to the ASPP and Decoder modules for feature extraction, respectively, after the image has traversed the backbone network. The ASPP module extracts and then merges the deep features. After applying a 1 – 1 convolution to adjust the number of channels, it is fed into the decoder.

The second decoder uses deep features from the encoder, which are upsampled 4 times, then merged with shallow features downsampled using 1 – 1 convolution. Subsequently, a 3 – 3 convolution is applied to further fuse the features. To obtain results that are the same size as the initial image, a bilinear interpolation approach is used to perform four-fold upsampling.

As a loss, our model employs a combined Lovász cross-entropy loss, thus combining both loss function properties. The idea is to leverage the benefits of both losses: the Lovász loss property of handling pixel-level segmentation tasks in severely imbalanced datasets, while cross-entropy captures pixel-wise classification accuracy. The loss function is further adjusted to address class imbalance in the dataset, using inverse square root frequency to assign weights to classes based on their frequencies, thereby enhancing the model's performance on minority classes. This technique further penalizes the model for errors in minority classes and scales the loss associated with each class. This helps to alter the impact of each class throughout training. This smoothed weighting ensures a better gradient flow and stable optimization. This can be particularly effective in datasets with extreme class imbalance, as it avoids overcompensating for rare classes.

### 3.2 Loss functions for segmentation

In addition to the traditional cross-entropy (Yeung et al., 2022) loss function, the current study carried out several trials using advanced loss functions that address the problem of data imbalance, such as Dice (Kato and Hotta, 2024) and Lovász (Berman et al., 2018) loss functions. An overview of the loss functions utilized in picture segmentation is provided in Table 1.

**Table 1:** Loss functions for segmentation utilized during our experimentation.

Loss functions	Application
Cross-entropy loss	Suitable for pixel-by-pixel classification tasks where every pixel belongs to one of multiple classes.
Dice loss	Useful for measuring the gap between ground truth and predicted segmentation, particularly in cases of class imbalance.
Lovász loss	Applied in semantic segmentation tasks, effectively managing pixel imbalance across multiple classes.
Combined loss	Balances recall and precision by combining multiple losses. For example, combining cross-entropy and Dice loss manages multi-class and binary segmentation.

#### 3.2.1 Cross-entropy loss

With the DeepLabV3Plus model, the default function is the cross-entropy loss. Cross-entropy loss measures the difference between each pixel's actual label across all classes and its predicted probability. The negative log-likelihood of the probability of the correct class at each pixel is known as the cross-entropy loss in multi-class segmentation. It is expressed as follows (Yeung et al., 2022):


$$\begin{array}{l} L_{\text{CE}}=-\overset{C}{\underset{c=1}{\sum }}\frac{1}{N}\overset{N}{\underset{i=1}{\sum }}y_{i,c}\log\left(p_{i,c}\right) \end{array}$$ (1)

where *C* is the number of classes, *y*_*i, c*_ is the ground truth label for the pixel *i* belonging to class *c* (usually one-hot encoded), *p*_*i, c*_ is the predicted probability for the pixel *i* belonging to class *c*, *N* is the total number of pixels.

#### 3.2.2 Lovász loss

The Lovász Loss is intended to directly optimize the IoU measure, offering a smooth and differentiable substitute for the Jaccard index that is appropriate for gradient-based learning (Berman et al., 2018). The Lovász Loss is defined mathematically using a convex Lovász extension of the sub-modular Jaccard index, given a set of predictions and the ground truth labels that correspond to them. The Lovász loss is defined as:


$$\begin{array}{l} L_{\text{Lovász}}=\frac{1}{\left|C\right|}\underset{c\in C}{\sum }{\bar{\Delta }}_{J_{c}}\left(m\left(c\right)\right) \end{array}$$ (2)

Here, *C* denotes the set of classes, **m**(*c*) represents the vector of pixel-wise prediction errors for class *c*, sorted in descending order, and ${\bar{\Delta }}_{J_{c}}$ denotes the gradient of the Jaccard index (Lovász extension) for class *c*.

#### 3.2.3 Dice loss

Dice loss measures the similarity between the anticipated segmentation mask and the ground truth mask in segmentation tasks (Kato and Hotta, 2024). It is based on the Dice Similarity Coefficient, also known as the Sørensen-Dice index. When datasets have unequal class distributions, such as in healthcare imaging, where the areas of focus are usually substantially smaller than the background, this coefficient is especially helpful. The Dice loss is calculated as follows:


$$\begin{array}{l} L_{\text{Dice}}=1-\frac{2\overset{N}{\underset{i=1}{\sum }}y_{i}{ŷ}_{i}}{\overset{N}{\underset{i=1}{\sum }}y_{i}+\overset{N}{\underset{i=1}{\sum }}{ŷ}_{i}+\epsilon } \end{array}$$ (3)

Where *N* is the total number of pixels, *y*_*i*_ is the ground truth label for pixel *i*, and ŷ_*i*_ is the predicted label for pixel *i*. A small constant ϵ is added in the denominator to avoid division by zero.

### 3.3 Class imbalance strategies

To address class imbalance in tasks such as image segmentation, different strategies are employed to allocate class weights to enhance the model's performance on minority classes. To penalize the model for mistakes in minority classes, these techniques scale the loss per class, helping adjust the effect of each class during training. Some of the most popular weighting techniques used are discussed below.

The minority classes are given larger weights via the inverse frequency approach. The overall number of pixels that belong to a class is inversely proportional to its class weight. As a result, the class with fewer pixels will be given greater weight, motivating the model to focus more on it.


$$\begin{array}{l} w_{c}=\frac{1}{f_{c}} \end{array}$$ (4)

where *w*_*c*_ is the weight of class *c*. *f*_*c*_ is the frequency of class *c* in the dataset (e.g., the number of pixels or samples).

Using the median frequency of all classes, the median frequency approach determines a class weight. A more reliable indicator of central tendency than the mean, the median frequency is less prone to outliers. This method normalizes each class's weight relative to the class with the median frequency of occurrences. The weight for class *c* using the Median Frequency approach is given by:


$$\begin{array}{l} w_{c}=\frac{\text{median}\left(f_{1},f_{2},\ldots ,f_{C}\right)}{f_{c}} \end{array}$$ (5)

where *w*_*c*_ is the weight of the class *c*. *f*_*c*_ is the frequency of class *c* (e.g., the number of pixels or samples in class *c*). median(*f*_1_, *f*_2_, …, *f*_*C*_) is the median frequency of all classes.

The weights are improved using the inverse square root frequency technique by taking the inverse square root of the class frequencies. Compared to the inverse frequency method, this approach lessens the impact of very small class frequencies, giving minority classes a more moderate emphasis. The weight for class *c* using the inverse square root frequency approach is given by:


$$\begin{array}{l} w_{c}=\frac{1}{\sqrt{f_{c}}} \end{array}$$ (6)

where *w*_*c*_ is the weight of class *c*. *f*_*c*_ is the frequency of class *c* (e.g., the number of pixels or samples in class *c*).

### 3.4 Experimental setup

Our tests were carried out using open-source PyTorch-based MMSegmentation tools for semantic segmentation. It is a standardized framework for applying, evaluating, and comparing semantic segmentation methods. It works well with many different kinds of semantic segmentation models. All tests were conducted in Python 3.10.12 using Google Colab, with the 0.30.0 MMSegmentation toolkit and 1.6.0 mmcv-full packages. The number of training iterations was set at 200.

Images from campaign 2, captured by a UAV, were used for model training, while images from campaigns 3–8 were reserved for testing. Imagery from campaign 1 was excluded due to mislabeled masks. As loss functions, we employed Dice loss, Lovász loss, and cross-entropy loss, as well as their various combinations. To improve the model's performance on minority classes, we used techniques such as inverse frequency, median frequency, and inverse square root frequency to assign class weights. Furthermore, a number of evaluation metrics were used, including accuracy (Acc), mean accuracy (mAcc), average accuracy (aAcc), intersection over union (IoU), and mean intersection over union (mIoU). Due to hardware and software limitations, semantic segmentation cannot be applied to the high-resolution dataset utilized in this study. Therefore, for training and testing, we cropped non-overlapping 480 × 352 resolution patch images. The class weights initialized by various class weighting strategies during our experiments are mentioned in Table 2.

**Table 2:** Class weighting strategies utilized and weights assigned during our experimentation.

Label	Class	Inverse frequency	Median frequency	Inverse square root frequency
0	Background	1.517	0.299	1.232
1	Tobacco	6.928	1.366	2.634
2	Weed	5.080	1.000	2.253

#### 3.4.1 Dataset

**Figure 2:**
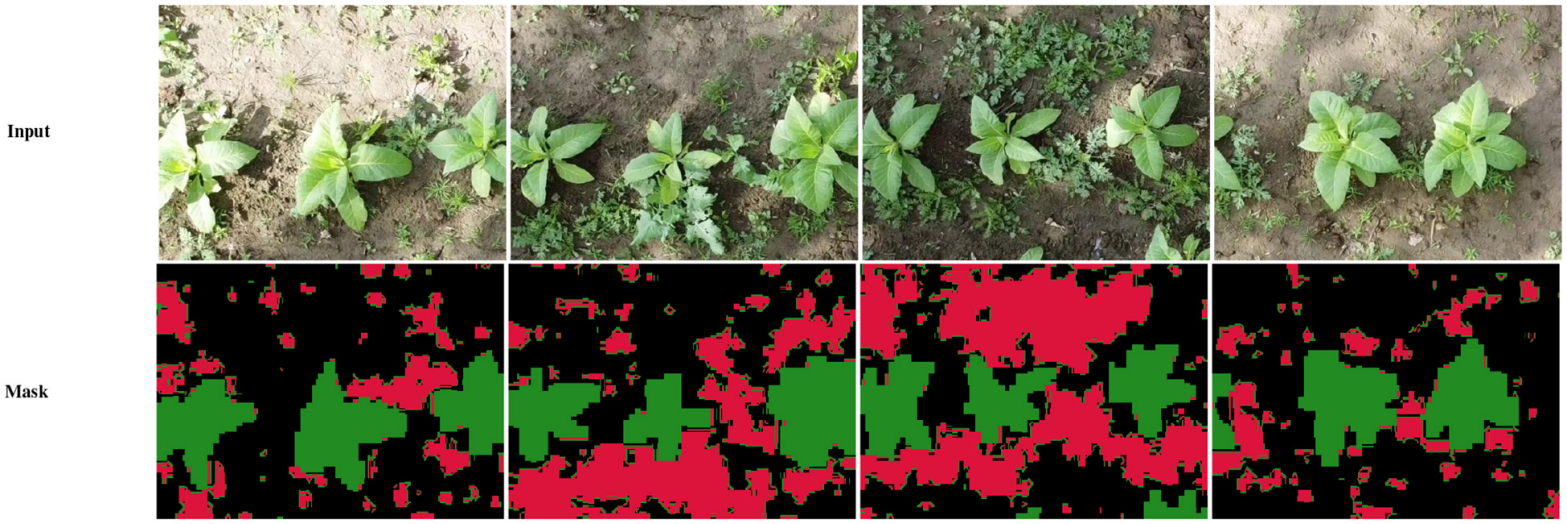
Examples of tobacco weed UAV image dataset samples: the RGB images in the first row are the input images, and the second row shows their corresponding mask images.

A publicly available tobacco-weed UAV-based dataset (Moazzam et al., 2023) was used to evaluate the proposed model. Eight tobacco crop farms have been captured at varying phases of growth, with crop ages ranging from roughly 15–40 days as seen in Table 3. The original resolution of the captured images was 1,920 × 1,080 pixels; however, preprocessed images of 480 × 352 resolution as seen in Figure 2 were used for efficient processing.

**Table 3:** Details regarding UAV-based tobacco weed dataset.

Campaign	Images No.	Time/date	Soil condition
1	864	2:30 pm/9 April 2021	After irrigation
2	936	3:30 pm/7 April 2021	Before irrigation
3	120	3:38 pm/7 April 2021	After irrigation
4	120	5:52 pm/7 April 2021	Before irrigation
5	120	6:33 pm/7 April 2021	After irrigation
6	120	3:27 pm/9 April 2021	After irrigation
7	120	3:43 pm/9 April 2021	Before irrigation
8	120	3:59 pm/9 April 2021	After irrigate

Images from one field were utilized for training, while images from the remaining seven fields were used for testing to determine how well the model performs. Table 1 enumerates these eight drone fly campaigns. The dataset was obtained with a ground sampling distance of 0.1 cm/pixel and has an average altitude of 4 m. Each pixel is labeled as 0 for background/soil, 1 for crop, and 2 for weed. For color mask visualization, black, green, and red are used to represent soil, crops, and weeds, respectively. However, the dataset is class imbalanced where background has 65.86%, crop has 14.43% and the weed has 19.71% pixel count as seen in Table 4.

**Table 4:** Tobacco weed dataset class pixel count distribution.

Label	Class	Pixel count	Percentage
0	Background	104,163,282	65.87%
1	Tobacco	22,813,874	14.43%
2	Weed	31,169,404	19.71%

#### 3.4.2 Evaluation metrics

The following metrics are utilized to assess the performance of the segmentation models: mIoU, IoU, aAcc, and mAcc.

The mean accuracy (mAcc) is the average class-wise pixel classification accuracy over all classes. In semantic segmentation, it is computed as the average of per-class accuracies, where each class's accuracy is defined as the ratio of correctly classified pixels in that class to the total number of ground-truth pixels in that class. Thus, mAcc can be considered as the average class-wise recall/sensitivity and can be defined as follows:


$$\begin{array}{c} \text{mAcc}=\frac{1}{C}\overset{C}{\underset{c=1}{\sum }}\frac{{\text{TP}}_{c}}{{\text{TP}}_{c}+{\text{FN}}_{c}} \end{array}$$ (7)

where *TP*_*c*_ is the number of correctly categorized positive pixels, or true positives for class *c*, *FN*_*c*_ is the number of positive instances that were incorrectly predicted as negative for class *c*, and *C* is the total number of classes.

The mIoU, also known as the Jaccard similarity coefficient, is computed by averaging the IoU scores for each data class across all images. FP is penalized by this statistical measure of precision, to put it simply. The IoU (or Jaccard) metric has the following mathematical definition:


$$\begin{array}{c} \text{mIoU}=\frac{1}{C}\overset{C}{\underset{c=1}{\sum }}\frac{{\text{TP}}_{c}}{{\text{TP}}_{c}+{\text{FP}}_{c}+{\text{FN}}_{c}} \end{array}$$ (8)

where *TP*_*c*_ is the number of true positives for class *c*, *FN*_*c*_ is the number of false negatives for class *c*, *FP*_*c*_ is the number of false positives for class *c*, and *C* is the total number of classes.

The term aAcc usually refers to the mean of individual accuracies for several classes or data subsets. When you want to check how well the model performs on each class separately, without favoring the majority class, or when assessing performance on imbalanced datasets, it can be helpful. The average accuracy formula is as follows:


$$\begin{array}{c} \text{aAcc}=\frac{1}{N}\overset{N}{\underset{i=1}{\sum }}{\text{Acc}}_{i} \end{array}$$ (9)

Where *N* is the total number of classes or samples. Acc_*i*_ is the accuracy for the *i*-th class or sample.

It is worth noting that this study did not specifically report precision, recall, or F1-score. Although overall accuracy and mIoU provide partial information about the prediction quality, they do not isolate false-positive errors. This is recognized as a limitation and limits a thorough analysis of class-specific false positives. In the future, we will use precision, recall, and F1-score to provide a more comprehensive evaluation.

## 4 Results

This section presents the results of various baseline deep learning models (evaluated using their default configurations) on different field datasets, compares them using different loss functions and class-imbalance techniques, and discusses the algorithm's limitations relative to the proposed model. All comparisons are reported using quantitative results.

### 4.1 Model comparisons

The overall results of the five selected models utilizing cross-entropy loss and the proposed model are compared. These models are trained and evaluated on the Tobacco Weed dataset, with results presented in Table 5. All models are trained on imagery from campaign two, and their performance and robustness are tested on the remaining campaign images.

**Table 5:** Performance comparison between various deep learning pre-trained models and the proposed model, trained/tested on different campaigns of the tobacco weed UAV-based dataset. All evaluation metrics are reported in percentage (%), with the best-performing model and its corresponding values highlighted in bold.

Models	Metrics	Train C2	Test C3	Test C4	Test C5	Test C6	Test C7	Test C8	Mean ±SD
PSPNet	aAcc	88.70	92.12	93.66	93.96	92.54	91.84	93.17	92.88 ± 0.86
	mIoU	70.89	78.22	82.68	84.30	66.35	74.33	71.93	76.30 ± 6.52
	mAcc	80.31	86.98	89.31	90.18	70.17	81.20	77.26	82.51 ± 7.35
DeepLabV3 + ResNet50	aAcc	90.31	94.15	95.05	95.18	93.48	93.63	94.70	94.37 ± 0.72
	mIoU	73.22	82.26	85.68	87.01	70.28	79.17	77.63	80.34 ± 5.93
	mAcc	81.79	89.23	90.82	91.54	73.48	85.20	82.18	85.41 ± 6.33
ANN ResNet50	aAcc	88.93	92.26	93.89	94.13	93.28	92.17	93.48	93.20 ± 0.67
	mIoU	71.28	78.44	83.13	84.58	69.02	74.95	72.63	77.13 ± 6.26
	mAcc	80.32	87.23	89.58	90.25	72.95	82.58	78.64	83.54 ± 6.82
UPerNetResNet50	aAcc	90.04	93.47	94.83	94.88	93.31	92.90	94.14	93.92 ± 0.78
	mIoU	73.07	80.81	85.28	86.25	68.32	76.71	74.62	78.67 ± 6.43
	mAcc	81.46	88.25	90.56	90.97	71.78	83.13	79.53	84.04 ± 7.08
DeepLabV3+ResNeSt	aAcc	91.19	95.00	96.17	96.20	94.65	94.36	95.47	95.30 ± 0.72
	mIoU	75.13	84.37	88.65	89.58	74.42	80.92	79.87	82.96 ± 5.59
	mAcc	83.09	90.79	93.11	93.73	77.31	86.55	84.19	87.61 ± 6.20
Proposed model	aAcc	91.45	95.47	96.52	96.51	95.72	95.12	96.21	95.93 ± 0.58
	mIoU	75.60	85.39	89.66	90.36	78.58	83.16	82.76	84.99 ± 4.47
	mAcc	84.25	92.60	94.86	95.08	81.58	89.33	87.74	90.20 ± 5.15

The overall findings, which are summarized in Table 5, show that DeepLabV3Plus ResNeSt performs well during the training and testing phases compared to other benchmark methods. In training, DeepLabV3Plus ResNeSt achieved an accuracy rate of 91.19%, mIoU of 75.13%, and mAcc of 83.09%; in testing, it achieved an accuracy rate of 95.30%±0.72, mIoU of 82.96%±5.59 and mAcc of 87.61%±6.20, which is the average of all testing sessions. DeepLabV3Plus ResNet50, in contrast, came in second place behind DeepLabV3Plus ResNeSt. The results of the DeepLabV3Plus ResNet training phase showed an aAcc of 90.31%, an mIoU of 73.22% and an mAcc of 81.79%. Furthermore, it achieved an average aAcc of 94.37%±0.72, 80.34%±5.93 mIoU, and 85.41%±6.33 mAcc during the testing phase, which is the overall average of all campaigns used for testing. However, the DeepLabV3Plus models with ResNeSt and ResNet backbones, which act as feature extractors that pass feature maps through the rest of the network, fared better than other models, including PSPNET, ANN ResNet50, and UPERNET ResNet50.

However, Table 5 also shows that our proposed model outperformed all benchmark methods in both the training and testing phases. Our proposed improved DeepLabV3Plus ResNeSt model utilizes the Lovász cross-entropy combined loss and inverse square root frequency suggested class weights. Thus, this resulted in an aAcc of 91.45%, a mIoU of 75.60% and an mAcc of 84.25% in the training phase; Meanwhile, 95.93%±0.58 aAcc, 84.99%±4.47 mIoU, and 90.20%±5.15 mAcc were on average achieved during the testing phase. These results outperformed all other benchmark models.

**Figure 3:**
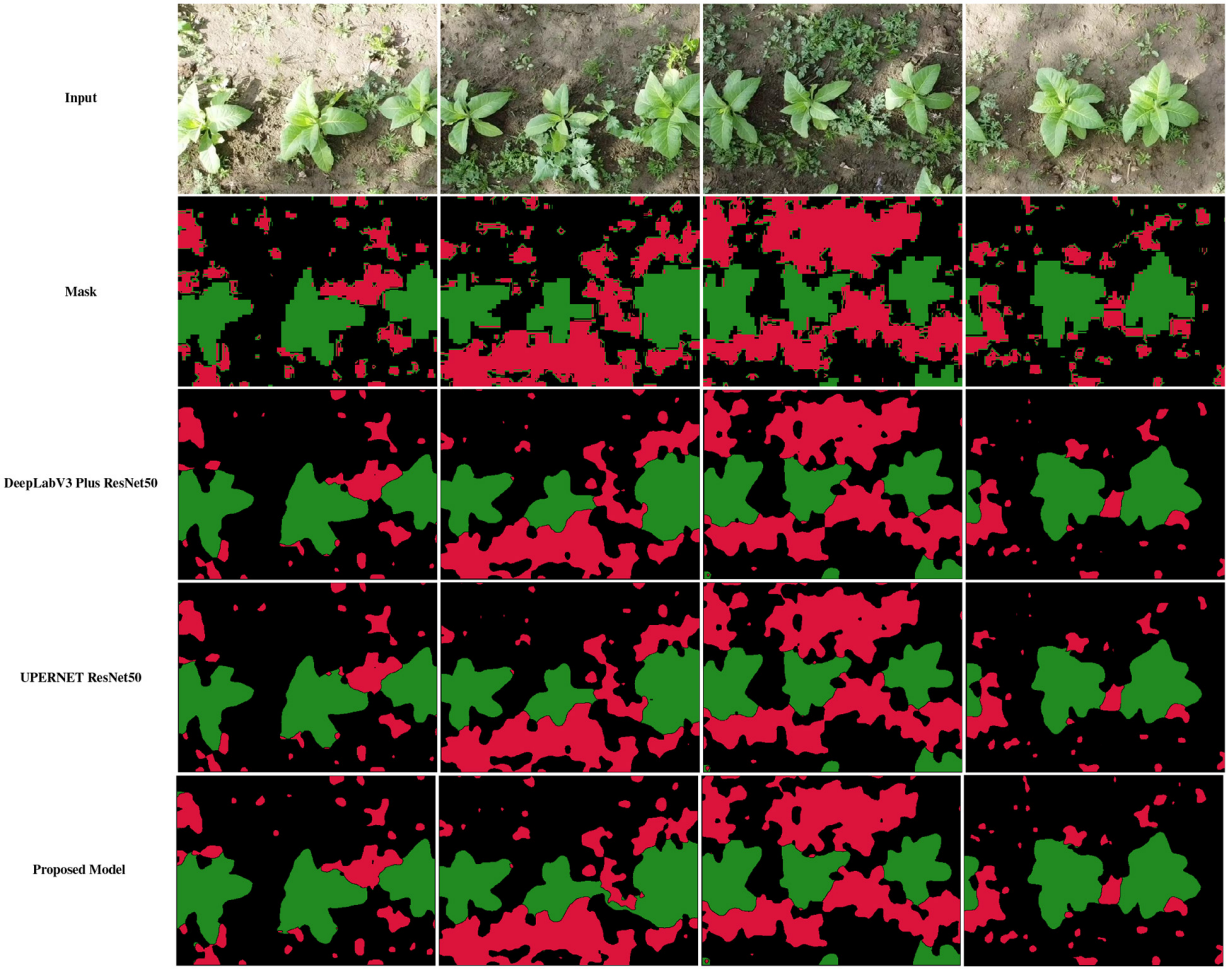
Visual comparison among best performing deep learning models and our proposed model for weed detection, trained/tested on different campaigns of tobacco weed UAV-based dataset.

The main reason for the proposed model's exceptional performance is the numerous enhancements in DeepLabV3Plus, which greatly improve its ability to perform pixel-wise segmentation, especially in complex scenes, as shown in Figure 3. The robust Atrous Convolution, also known as dilated convolution, is combined with a decoder module in DeepLabV3Plus to improve segmentation accuracy and refine borders. Conversely, our proposed model uses DeepLabV3Plus with ResNeSt as the backbone, which outperformed it. The performance boost is due to ResNeSt's split-attention block, which, unlike ResNet, enables more detailed feature extraction and fine-grained segmentation. High-resolution images or tasks that require intricate feature interactions might be particularly challenging for the basic ResNet architecture to represent fine-grained features or simulate more complex patterns.

### 4.2 Comparison of loss function and class imbalance handling

The overall results of our proposed model and the DeepLabV3Plus ResNeSt model with nine different loss functions introduced are presented in Table 6. The loss functions utilized include Lovász, Dice, cross-entropy, and combined loss functions to determine the loss that is best optimized for weed detection. All scores are expressed as a percentage (%).

**Table 6:** Performance comparison between various loss functions utilized with DeepLabV3Plus ResNeSt model and proposed model, trained/tested on different campaigns of the tobacco weed UAV-based dataset. All evaluation metrics are reported in percentage (%), with the best-performing model and its corresponding values highlighted in bold.

Loss functions	Metrics	Train C2	Test C3	Test C4	Test C5	Test C6	Test C7	Test C8	Mean ±SD
Lovász loss	aAcc	90.76	94.43	95.71	95.75	94.62	94.16	95.23	94.98 ± 0.68
	mIoU	74.50	83.28	87.61	88.56	74.39	80.87	79.52	82.37 ± 5.30
	mAcc	82.88	90.57	93.11	93.62	77.65	86.68	84.16	87.63 ± 6.12
Dice loss	aAcc	90.21	93.40	95.17	95.28	94.73	94.18	95.45	94.70 ± 0.79
	mIoU	73.53	79.50	86.51	87.57	75.49	80.89	80.79	81.79 ± 4.53
	mAcc	83.28	91.23	93.91	94.04	78.81	88.02	86.59	88.77 ± 5.74
Cross-entropy loss	aAcc	91.17	94.95	96.12	96.24	94.55	94.30	95.35	95.25 ± 0.80
	mIoU	75.07	84.28	88.42	89.68	73.94	80.80	79.47	82.77 ± 5.91
	mAcc	82.93	90.47	92.76	93.65	76.83	86.31	83.76	87.30 ± 6.38
Dice + Lovász loss	aAcc	91.09	94.78	96.22	96.24	95.10	94.70	95.82	95.48 ± 0.70
	mIoU	75.01	83.44	88.86	89.70	76.37	81.92	81.47	83.63 ± 4.99
	mAcc	83.80	92.08	94.14	94.50	79.36	88.12	86.47	89.11 ± 5.76
Lovász + Dice loss	aAcc	91.17	94.85	96.23	96.25	94.99	94.80	95.84	95.49 ± 0.69
	mIoU	75.15	83.53	88.81	89.66	75.89	82.38	81.62	83.65 ± 5.07
	mAcc	83.66	91.86	93.82	94.24	78.88	88.20	86.36	88.89 ± 5.81
Lovász + Cross-entropy loss	aAcc	91.39	95.24	96.39	96.47	95.10	94.70	95.80	95.62 ± 0.72
	mIoU	75.56	85.07	89.22	90.24	76.02	81.94	81.00	83.92 ± 5.74
	mAcc	83.49	91.38	93.58	94.18	78.90	87.57	85.54	88.53 ± 5.88
Cross-entropy + Lovász loss	aAcc	91.32	95.10	96.35	96.43	94.52	94.41	95.44	95.38 ± 0.87
	mIoU	75.41	84.69	89.15	90.09	73.87	81.02	79.70	83.09 ± 6.24
	mAcc	83.17	90.76	93.21	93.85	76.68	86.42	84.03	87.49 ± 6.24
Cross-entropy + Dice loss	aAcc	91.28	94.92	96.37	96.44	95.01	94.73	95.81	95.55 ± 0.76
	mIoU	75.35	83.43	89.14	90.08	75.84	81.80	81.07	83.56 ± 5.52
	mAcc	83.49	91.55	93.84	94.25	78.73	87.76	85.83	88.66 ± 5.85
Dice cross + Entropy loss	aAcc	91.26	94.95	96.39	96.44	94.98	94.81	95.87	95.57 ± 0.75
	mIoU	75.32	83.79	89.27	90.13	76.02	82.18	81.44	83.81 ± 5.46
	mAcc	83.50	91.57	93.92	94.30	78.84	87.91	86.00	88.76 ± 5.81
Proposed model	aAcc	91.45	95.47	96.52	96.51	95.72	95.12	96.21	95.92 ± 0.58
	mIoU	75.60	85.39	89.66	90.36	78.58	83.16	82.76	84.99 ± 4.48
	mAcc	84.25	92.60	94.86	95.08	81.58	89.33	87.74	90.20 ± 5.15

The results show that DeepLabV3Plus with ResNeSt utilizing Lovász cross-entropy combined loss gives promising results compared to other benchmark loss functions. However, our proposed model still outperforms it. The model utilizing Lovász cross-entropy combined loss gave the best mean testing aAcc of 95.62%±0.78, and the best mean mIoU of 83.92%±5.74 , but a suboptimal mAcc of 88.53%±5.88 among all loss functions when utilized with the DeepLabV3Plus ResNeSt model.

The reason for such good performance in both aAcc and mIoU results when using combined Lovász and cross-entropy loss is the combination of both loss function properties. Here both structural consistency (measured by Lovász loss) and pixel-wise classification accuracy (measured by cross-entropy) play a crucial role. The goal is to capitalize on both loss advantages: Pixel-level segmentation tasks that deal with highly imbalanced datasets require the optimization of the boundaries and structure of the segmentation output, which is achieved by Lovász loss, while cross-entropy captures pixel-wise classification accuracy.

Additionally, DeepLabV3Plus ResNeSt, when utilized with the Dice Lovász loss, gives a mediocre mean aAcc of 95.48%±0.70 and mean mIoU of 83.63%±4.99 compared to when Lovász cross-entropy combined loss is used. This gives a promising mean mAcc of 89.11%±5.76 compared to all other loss functions. The aAcc for each class is impacted by the model's ability to anticipate each class. Both Dice loss and Lovász loss help to improve this. For underrepresented classes, Dice Loss maximizes overlap, thereby directly increasing per-class pixel accuracy. Lovász Loss, meanwhile, enhances the segmentation boundaries; objects are segregated more precisely, lowering false positives and false negatives and increasing the anticipated segmentation's accuracy.

**Figure 4:**
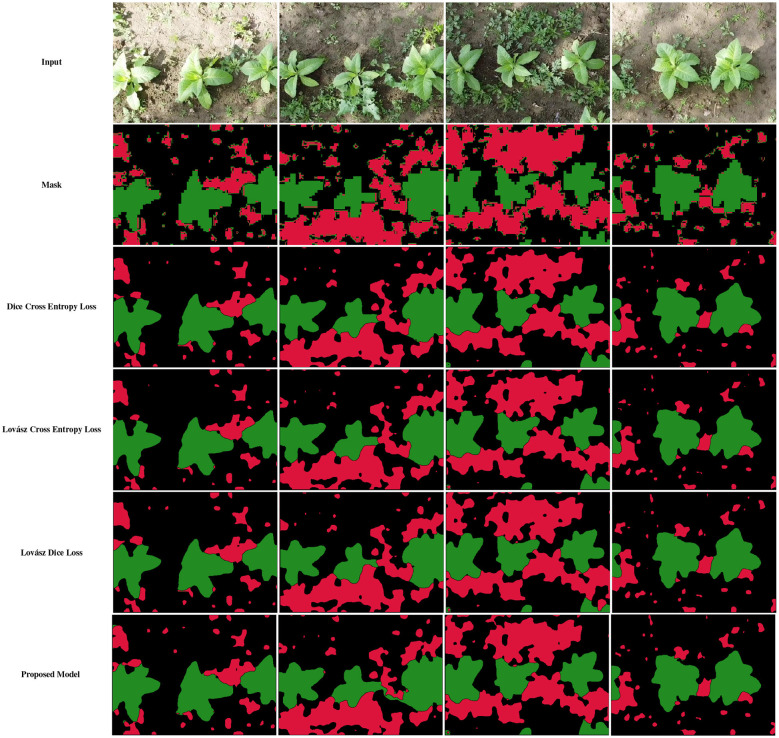
Visual comparison among best performing loss function models and the proposed model using DeepLabV3Plus ResNeSt for weed detection.

However, mIoU is generally used to report performance in weed-detection segmentation tasks in recent studies (Kong et al., 2024). As it evaluates how well the predicted object masks overlap with the ground truth, which is directly related to the quality of weed segmentation, as seen in Figure 4. Thus, we can safely claim that DeepLabV3Plus ResNeSt with Lovász cross-entropy combined loss achieves the best results among other loss functions, except for our proposed model.

Table 7 presents a comparison of the overall results for DeepLabV3Plus ResNeSt using Lovász cross-entropy loss functions with different class imbalance handling approaches and our proposed approach. We compared the proposed weed-detection model using various class imbalance techniques based on inverse and median frequencies.

**Table 7:** Performance comparison among various class weight models and the proposed model utilized with DeepLabV3Plus ResNeSt using Lovász cross-entropy loss, trained/tested on different campaigns of the tobacco weed UAV-Based dataset. All evaluation metrics are reported in percentage (%), with the best-performing model and its corresponding values highlighted in Bold.

Class weight models	Metrics	Train C2	Test C3	Test C4	Test C5	Test C6	Test C7	Test C8	Mean ±SD
Inverse frequency	aAcc	90.98	94.51	96.26	96.28	96.15	94.78	95.86	95.64 ± 0.79
	mIoU	74.68	81.52	88.57	89.53	80.76	81.87	81.54	83.97 ± 3.97
	mAcc	84.65	92.36	94.89	95.18	84.48	90.27	89.61	91.13 ± 3.98
Median frequency	aAcc	90.93	94.85	95.93	95.98	95.69	94.88	96.00	95.56 ± 0.55
	mIoU	74.75	82.91	87.82	89.04	78.98	82.45	82.20	83.90 ± 3.79
	mAcc	84.74	93.18	94.99	95.40	82.39	89.82	88.23	90.67 ± 4.95
Proposed model (Inverse Square Root Frequency)	aAcc	91.45	95.47	96.52	96.51	95.72	95.12	96.21	95.93 ± 0.58
	mIoU	75.60	85.39	89.66	90.36	78.58	83.16	82.76	84.99 ± 4.48
	mAcc	84.25	92.60	94.86	95.08	81.58	89.33	87.74	90.20 ± 5.15

When class weights are handled using the inverse square root frequency, as indicated in Table 7, the findings demonstrate that the proposed DeepLabV3Plus with ResNeSt, employing the combined Lovász cross-entropy loss, yields the most optimal results. The proposed model uses an inverse square root frequency and achieves the highest mean mAcc, mIoU, and aAcc testing results 95.93%±0.58, 84.99%±4.48, and 90.20%±5.15, respectively. Due to its more balanced approach to handling class imbalance, the proposed model that uses inverse square root frequency outperformed both the inverse frequency and the median frequency.

**Figure 5:**
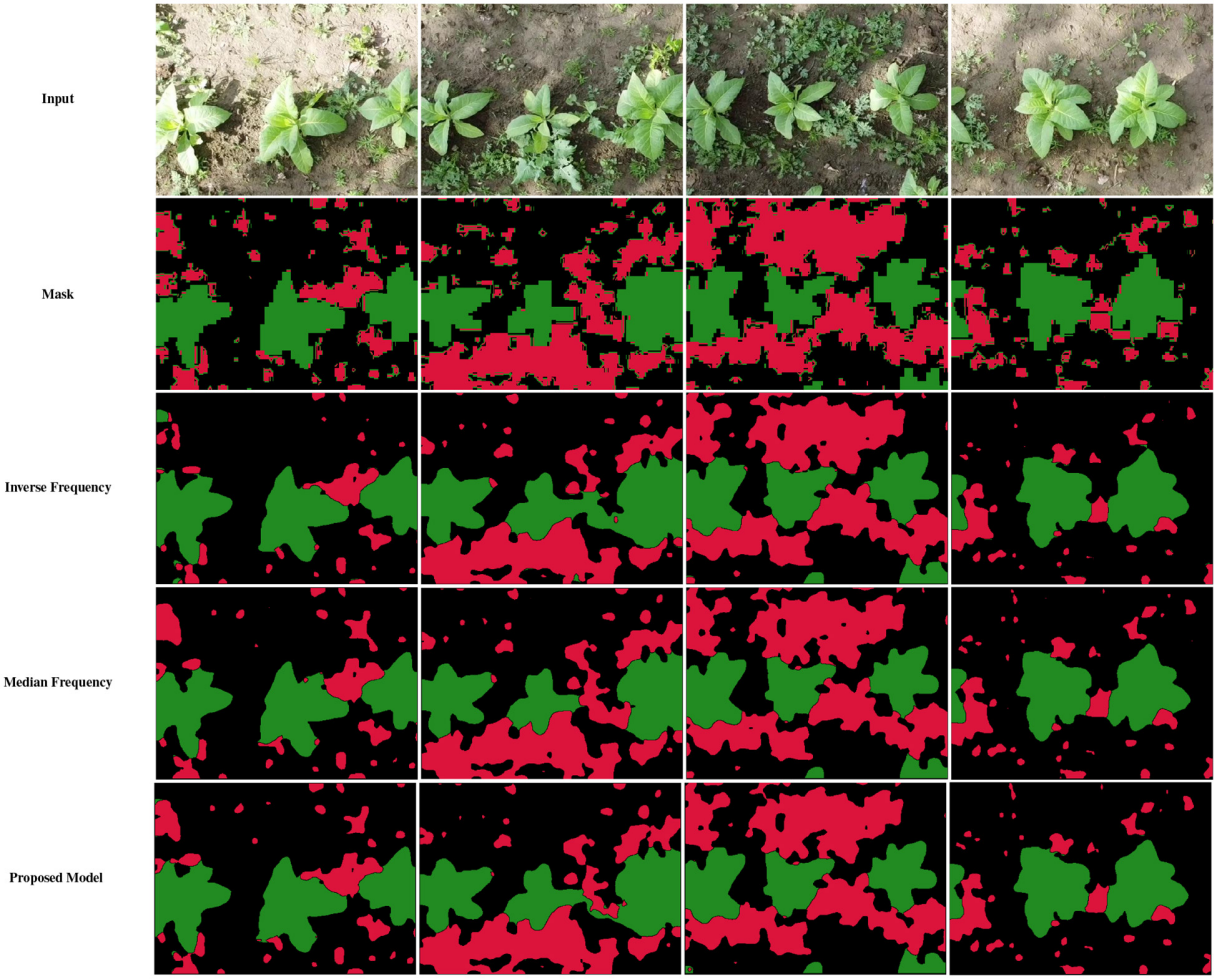
Visual comparison between various class weights models and proposed model using DeepLabV3Plus ResNeSt and Lovász cross-entropy loss for weed detection.

The proposed framework, utilizing the inverse square root frequency, was able to improve DeepLabV3Plus ResNeSt by using the capacity of Lovász cross-entropy loss to generalize across all classes. This was achieved by using the inverse square root of class frequencies, which reduces the risk of over-penalizing small classes while still giving them more weight than larger classes. This makes the proposed model a more reliable and effective choice for many segmentation tasks involving imbalanced datasets in real-world settings, as shown in Figure 5.

## 5 Discussion

The experimental results show that the proposed model performs better than other standard models across most metrics. The excellent performance of the proposed model comes from the effective combination of its three key parts. First, using ResNeSt as the main network, along with its split-attention blocks, enables more detailed feature extraction than regular ResNet models. This helps the model better recognize small details that are important for distinguishing weeds from crops and soil. Second, the combined Lovász cross-entropy loss function helps balance accurate pixel classification with maintaining the correct structure in the results. While cross-entropy ensures that each pixel is labeled correctly, the Lovász part focuses on improving the IoU metric, which better reflects the quality of the segmentation. Third, the inverse square root frequency class weighting helps address class imbalance by giving sufficient attention to less common weed classes without allowing the more common background class to dominate the learning process.

Regarding comparisons with other similar weed segmentation models, previous studies (Yu et al., 2022; Bak et al., 2023; Islam et al., 2025) based on DeepLabV3Plus achieved good mIoU scores: 91.53% for weed segmentation in soybean fields and 76% in maize fields. The proposed model achieved a lower mIoU than these models. The low mIoU of the proposed framework is attributed to the fact that the target crop type is different. In this study, we evaluate the framework for tobacco crops, whereas previous studies focus on soybean and maize. Soybean and maize fields differ from tobacco fields in plant morphology, canopy structure, crop–weed spatial distribution, and background characteristics. Weeds in this study are in different growth stages, often obscured by tobacco plants, and are captured under a variety of soil moisture, lighting, and irrigation conditions during different UAV campaigns for the tobacco-weed dataset. All these factors pose a significant challenge to accurate segmentation. Therefore, the mIoU obtained in this study should be viewed in light of the more complex tobacco-weed segmentation scenario, rather than as a direct loss or degradation in performance compared to other crop studies. Despite the above-mentioned difficulties, the proposed DeepLabV3Plus-ResNeSt framework achieved competitive performance for tobacco weed segmentation in UAV images, with a mean mIoU value of 84.99%, which is satisfactory for this task, especially in the case of class imbalance, complex background, and vegetation.

However, several important limitations should be considered. The performance of the proposed model varied across different images from the testing campaigns. This shows that the model is sensitive to environmental factors, such as different weather conditions, plant growth stages, and lighting conditions. This means that a model trained on data from a specific time or location might not work well in other situations without some extra adjustments. Also, even though the model performs well, the ResNeSt backbone requires significant computing power, which could make it hard to use on small, low-powered devices like those found on drones.

Another limitation of the present evaluation is that precision was not reported. Evaluation metrics, mIoU and mAcc give sufficient information about the segmentation performance; however, these metrics cannot provide much information about the false positives. The mIoU metric is affected by both false positives and false negatives, whereas mAcc primarily measures the model's accuracy in correctly identifying ground-truth pixels for each class. These metrics, therefore, provide an overall indication of segmentation quality. The proposed model shows improved class-wise segmentation performance and better overlap with the ground truth masks, with the higher mIoU and mAcc values than the baseline models. This is an indirect measure of the fact that the proposed model is likely to have fewer overall segmentation errors, false positives, and false negatives.

From a practical point of view, the model's excellent performance has significant implications for precision farming. More accurately separating weeds allows for the precise use of herbicides, which can lower chemical use and reduce costs. It also helps to protect the environment. The model consistently outperforms others across various campaigns, suggesting it could be a reliable tool for automated weed-tracking systems throughout the growing season.

## 6 Conclusion

Weeds in agricultural land have long been an issue for farmers. They constantly compete for resources on cultivated land. In addition to causing harm, these undesirable plants can make harvesting even more challenging (Kumar et al., 2024). These unwanted weeds will indirectly increase the cost of production, decrease the quality of the crop, increase the risk of pest and disease attacks, and decrease the economic worth of the cultivated land (Rao et al., 2020). Nowadays, smart agriculture includes advanced segmentation models that use deep learning and UAV-based remote sensing to detect weeds.

To identify and locate weed zones, we present a model framework that uses the DeepLabV3Plus ResNeSt model. The suggested model, which was trained using the Lovász cross-entropy coupled Loss and inverse square root frequency weighted class, achieves a mean aAcc of 95.93%, a mean mIoU of 84.99%, and a mean mAcc score of 90.20%. Nevertheless, more similar aerial images might be segmented using the trained model. As it requires a great deal of human involvement, accuracy, and attention to detail, manual segmentation can be challenging and time-consuming. Due to the limited number of pixels identified as weeds, the aerial imagery is considered a limited dataset. Therefore, we adjusted class weights using the inverse square root frequency model, thereby simplifying the segmentation process.

Although the proposed model exhibits acceptable spatial-temporal generalization across several campaigns within the same farm, its adaptability to various geographical areas and crop types was not evaluated in this study and will be investigated in future studies. By enhancing the segmentation model's reliability, this study contributes to precision agriculture and enables more accurate, sustainable farming practices.

## Data Availability

The original contributions presented in the study are included in the article/supplementary material, further inquiries can be directed to the corresponding authors.
